# Intake, Digestibility, Ruminal Fermentation, and In Situ Disappearance of Bermudagrass Hay by Lactating Beef Cows Offered Corn or Hominy Feed as Supplements at Two Different Rates

**DOI:** 10.3390/ani13111845

**Published:** 2023-06-01

**Authors:** Christine C. Nieman, Zibani Madzonga, Ashley N. Young-Kenworthy, Kenneth P. Coffey

**Affiliations:** 1 USDA-ARS Dale Bumpers Small Farms Research Center, Booneville, AR 72927, USA; 2 Department of Agricultural Research, Francistown P.O. Box 10275, Botswana; 3 Simmons Foods, Springdale, AR 72734, USA; 4 Department of Animal Science, University of Arkansas, Fayetteville, AR 72701, USA

**Keywords:** hominy, beef cow, corn grain, intake, bermudagrass, digestibility

## Abstract

**Simple Summary:**

The decision to provide energy and protein sources to beef cows, offering low- or medium-quality forages, is critical to producers in terms of economics and animal production, particularly during gestation and lactation stages. While corn has been the predominant energy supplement, co-products of corn have increasingly been incorporated as energy supplements in forage-based diets because of lower cost. The objective of this study was to determine the effect of level of hominy feed supplementation on intake, digestibility, ruminal fermentation characteristics, and in situ dry matter disappearance of bermudagrass hay in lactating beef cows. Treatments were low hominy feed fed at 0.25% of body weight, medium hominy feed fed at 0.50% of body weight, low ground corn fed at 0.25% of body weight, medium ground corn fed at 0.50% of body weight, and no supplement (control). Hay dry-matter intake was not affected by supplementation and total dry-matter intake was greater with medium rates of supplementation. Supplementation did not affect ruminal parameters and did not affect dry matter disappearance of forages. Therefore, hominy feed can be used as an alternative feed to corn as an energy supplement without causing negative effects on measurements that are potential indicators of animal performance.

**Abstract:**

Hominy feed (HF) has been evaluated in feedlot and dairy rations but has not been evaluated as a supplemental energy source for lactating beef cows. The objective of this study was to determine the effect of level of HF supplementation on intake, digestibility, ruminal fermentation characteristics, and in situ dry matter (DM) disappearance of bermudagrass hay. Five ruminally cannulated lactating beef cows (body weight (BW) = 596 kg, SE = 13.9) were used in an experiment with a 5 × 5 Latin square design. Treatments were a bermudagrass hay-basal diet with low HF (LH; 0.25% BW), medium HF (MH; 0.50% BW), low ground corn (LC; 0.25% BW), medium ground corn (MC; 0.50% BW) and no supplement (CON). Cows were housed individually, and supplements were offered at 0800 daily. Hay was offered to maintain 10% refusal. Periods were 16–d, with 10 d for adaptation. Ruminal fluid was sampled on d 14 of each period for measurement of pH, volatile fatty acids, and rumen ammonia-N. An in situ degradation experiment for bermudagrass hay was completed for each diet. Hay dry-matter intake (DMI % BW) was not affected (*p* = 0.14) by supplement, but total DMI (DMI % BW) was greater (*p* ≤ 0.05) in MH and MC compared to LH and CON. Digestible DMI % BW was greater (*p* = 0.05) in MH compared to LC, MC was intermediate, and LH and CON were lesser (*p* ≤ 0.01) than all other diets. Dry-matter fill, passage rate, and retention time did not differ by diet (*p* ≥ 0.31). A diet × time interaction was observed for ammonia-N (*p* = 0.0002), and propionate (*p* = 0.02) time effects were observed for other parameters, but no diet effects. Bermudagrass hay’s potentially degradable fraction was greater (*p* ≤ 0.05) in LH than MH and CON, but effective DM degradability was not different (*p* = 0.39) among diets. Overall, no diets reduced hay intake or disappearance compared to CON; therefore, no negative associative effects were observed from any of the supplements at the levels offered in this study.

## 1. Introduction

The decision to provide energy and protein sources to beef cows, offering low- or medium-quality forages, is critical to producers in terms of economics and animal production, particularly in the winter or the dry season and during gestation and lactation stages. While corn has been the predominant energy supplement used over the years, co-products of corn have increasingly been incorporated as energy supplements in forage-based diets because of lower cost [1]. Additionally, co-products contain more fibrous components and lower non-structural carbohydrates, and therefore have fewer negative impacts on forage intake and digestibility, compared to high-starch supplements that can lower rumen pH and hinder fibrolytic bacteria [2]. Hominy feed is the byproduct of the separation of corn kernel components into flour, grits, and meal, in which the endosperm is partially separated from germ and pericarp by dry grinding [3]. Typically, hominy feeds contain a greater neutral detergent fiber (NDF), lesser starch, and greater fat than ground corn [4]. Hominy feed has been evaluated for cattle-finishing diets [4,5] and dairy diets [6,7,8], but limited research has been published about its potential use as a supplemental energy source for lactating beef cows on high-forage diets.

Concerns with supplementation for ruminants, offered via high-forage diets, have an effect on intake and digestibility, of which, these responses differ based on forage nutritive value and supplement type. Protein supplements (554 g/kg soybean meal, 446 g/kg corn gluten meal) resulted in greater intakes by lambs consuming low-crude protein (CP) forages (52 g/kg CP), while lambs consuming forage of 102 g/kg or 142 g/kg CP did not have an increased intake [9]. Similarly, Nieman et al. [10] observed no increases in intake with dried distillers grains with solubles (DDGS) supplementation when forage met the CP requirements of lactating beef cows. Without adequate CP or rumen-degradable protein (RDP), improvements in cow performance with starch supplementation are limited, and greater starch supplementation rates result in a reduced intake [11]. However, on diets of adequate or excessive CP availability, forage intake and digestibility may benefit from the additional energy provided by starch supplementation [8,12,13]. It is hypothesized that hominy feed will improve forage utilization relative to ground corn because of lower starch, and greater digestible fiber and fat content in the hominy feed. The objective of this study was to determine the effect of hominy feed supplementation on intake, digestibility, ruminal fermentation, and in situ disappearance of bermudagrass hay in lactating beef cows.

## 2. Materials and Methods

### 2.1. Animal Procedures

All procedures in the following experiment were approved by the University of Arkansas Institutional Animal Care and Use Committee (Protocol #1103).

Five multiparous, lactating, ruminally cannulated, fall-calving beef cows (body weight (BW) = 596 ± 13.9 kg) of predominantly Gelbvieh and Angus breeding were used in a study with a 5 × 5 Latin square design to compare 5 dietary treatments during 5, 16-d experimental periods. Cows were housed individually in 6.1 × 6.1 m pens with wood chip bedding. Each day, calves were allowed to nurse the cows at 0745 and 1630 h. Calves were removed immediately after nursing and did not consume any hay or supplement offered to the cows. At the end of each period, cows and calves were commingled and offered bermudagrass hay for 5 d in a dry lot to allow for exercise and rumen equilibrium across cows. Following the first period, cows were exposed to a bull for 21 d, then returned to the facility for the initiation of period 2. Feces and wet bedding material were removed twice daily.

Cows were offered a bermudagrass hay-basal diet along with either no supplemental concentrate (CON) or supplements of hominy offered at 0.25 (LH) or 0.50% of BW (MH), or corn offered at 0.25 (LC) or 0.50% of BW (MC) on an as-fed basis. Hay was offered to maintain a minimum of 10% refusal. Cows were weighed at the beginning and end of each period and the initial weight was used to determine the amount of supplement offered on a %BW basis. Cows were offered their respective supplement at 0800 h daily after orts were removed, and cows readily consumed the supplement in 20 min, leaving no supplement refusal. Water was supplied ad libitum and a commercial mineral supplement (110 g; Purina Wind and Rain All Season 4, Purina Mills, Gray Summit, MO, USA) was offered to each cow including the CON at 0800 daily.

Each period consisted of a 10-d dietary-adaptation period followed by a 5-d period of collecting fecal grab samples at 0800 and 1630 h. Samples of hay, supplement, orts, and fecal grab samples were taken daily and dried to a constant weight at 50 °C to determine DM. The hay and supplement samples collected daily were composited by weight within the period, resulting in a total of 5 samples each of hay, corn, and hominy feed. Ort and fecal samples were composited by weight for each animal within each period, resulting in a total of 25 samples each of orts and feces.

### 2.2. Evacuations and Passage Rates

Passage rate (k_p_) was estimated using total ruminal evacuation [14]. On d 16 of each experimental period, total ruminal evacuations were carried out immediately preceding the morning feeding (0730) and at 6 h after feeding. For each session, total ruminal contents were emptied into two lined plastic cans per cow, weighed, mixed thoroughly, then sampled, and the contents returned into the rumen with haste. Representative samples of ruminal contents were weighed into duplicate aluminum pans and dried to constant weight in a forced-air oven at 50 °C. Ruminal samples, and all feed and ort samples, were analyzed for acid-detergent insoluble ash (ADIA). The fractional passage rate of ADIA (k_p_) was determined by dividing the mean intake of ADIA (g/h) by the mean ruminal mass of ADIA (mean of 0 and 6 h samples). Ruminal retention time is calculated as the inverse of fractional-passage rate.

### 2.3. Ruminal Measurements

Ruminal fluid was sampled on d 14 of each period immediately prior to feeding, and 1, 3, 5, 7, 9, 11, and 13 h after the morning supplement feeding. Ruminal contents were collected from 4 different locations in the rumen and composited in a bucket. The composited sample was mixed, then strained through 4 layers of cotton cheesecloth into 120-mL plastic specimen containers. Ruminal pH was measured and recorded immediately using a portable pH meter (Denver AP5, Arvada, CO, USA). One milliliter of ruminal fluid was mixed with 200 µL of 12.5% meta-phosphoric acid, and frozen at −20 °C for later analysis of volatile fatty acids (VFA). Another 1 mL of ruminal fluid was mixed with 400 µL of 50% (*v*/*v*) hydrochloric acid and then frozen at −20 °C for later ammonia-N analysis.

### 2.4. Ruminal In Situ DM Disappearance

Dry-matter disappearance in the rumen was determined on representative samples of bermudagrass hay. Nylon bags (10 × 20 cm; 53 ± 15 µm porosity; ANKOM Technology Corp, Macedon, NY, USA) containing 5 g of ground (2 mm, Wiley Mill, Arthur H. Thomas, Philadelphia, PA, USA) bermudagrass hay were inserted in reverse order into the rumen on d 11 through d 16 for incubation times of 124, 100, 76, 52, 24, 16, 12, 8, and 4 h. All bags were removed simultaneously on d 16 at 2100 and placed in cold tap water to rinse off adhering particles and to inhibit any further microbial activity.

Bags containing bermudagrass hay (5 g) that were not incubated in the rumen (zero-hour bags), along with all incubated bags, were rinsed 10 times in a top-loading washing machine for 1 min with agitation in fresh tap-water, followed by a 2 min spin cycle. After rinsing, the bags were dried in a forced-draft oven at 50 °C for a minimum of 48-h. The dried sample bags were allowed to air equilibrate for a minimum of 72 h at room temperature, then weighed.

### 2.5. Chemical Analysis

Feed, ort, and fecal samples were ground to pass through a 1 mm screen using a Wiley mill (Arthur H. Thomas, Philadelphia, PA, USA). Nitrogen was measured on feed samples using the total combustion method (Elementar Americas Inc., Ronkonkoma, NY, USA method 990.03; [15]). Fat was determined using ether extraction (method 920.39; [15]) by the Central Analytical Laboratory at the University of Arkansas. Neutral detergent fiber and ADF were measured on all samples with the filter-bag procedure [16] using the ANKOM200/220 Fiber Analyzer (ANKOM Technology Corporation, Macedon, NY, USA). The NDF procedure included α-amylase, and the residue included residual ash. The ADF procedure was conducted on separate forage, hominy feed (HF), ground corn (GC), fecal, and ort samples that had not previously been solubilized with NDF solution. Residues from this ADF procedure were reduced to ash in a muffle furnace (forage, HF, GC, orts, and feces; method 942.05, [15]) to determine ADIA. Acid-detergent insoluble ash residues were used to calculate ADIA concentrations in the consumed diet and in the feces. These values were used to estimate the passage rate, as described above, and to estimate DM digestibility (g/kg) using the following equations:

DM digestibility = 100 − 100 × (Mfd/Mfc),

where Mfd = grams per kilogram ADIA in the feed; and Mfc = grams per kilogram ADIA in the feces.

Frozen ruminal fluid samples designated for VFA analyses were thawed overnight at room temperature, then agitated on a Vortex-Genie and centrifuged (2000× *g*) for 5 min. Volatile fatty acids were analyzed according to the procedures of Erwin et al. [17] using automated-gas chromatography (Hewlett Packard 5890 with automatic sample injector HP-7673, Avondale, PA, USA) fitted with a NukolTM-fused silica capillary column (30 m × 0.25 mm Ø × 0.25 µm film thickness (Supelco Inc., Bellefonte, PA, USA), a 5 m × 0.25 mm Ø. fused silica intermediate-polarity guard column (Supelco Inc.), and an FID detector.

The frozen ruminal-fluid samples designated for ammonia-N analysis were thawed, vortexed, and centrifuged similarly to those for VFA analysis. Ammonia-N concentrations were determined using the phenol–hypochlorite procedure [18] using a Shimadzu UV-VIS Spectrophotometer T1201S (Shimadzu, Inc., Kyoto, Japan).

### 2.6. Statistical Analysis

Intake, digestibility, and passage-rate data were analyzed using PROC GLIMMIX of SAS (SAS Institute Inc., Cary, NC, USA) for a 5 × 5 Latin square design. Diet was considered a fixed effect, and period and cow were considered random effects. The LSMEANS option was used to generate individual diet means. Significance was declared at *p* ≤ 0.05. When a significant difference was detected for diet, pairwise comparisons were tested using an F-protected *t*-test.

Fermentation data were analyzed using PROC GLIMMIX as a 5 × 5 Latin square tested for the effects of diet, sampling time, and their interactions. Diet and sampling time were considered fixed effects, and period and cow were considered random effects. Sampling time was used as a repeated measurement with cow within period as the subject. The LSMEANS option was used to generate individual treatment means. Significance was declared at *p* < 0.05. To help explain significant treatment effects, diet effects and diet × sampling time interactions were further separated within sampling time using pairwise F-protected *t*-tests.

The proportion of DM remaining in the in situ bags at each incubation time was fit to a nonlinear statistical model using PROC NLIN of SAS with lag following the model of Mertens and Loften [19]. The fraction that disappeared at a measurable rate (B), the disappearance lag time, the rate of DM disappearance (k_d_), and the undegradable fraction (U) were derived directly from the model, whereas the immediately soluble (water-soluble) fraction (A) was calculated as 100 − (B + U). Effective ruminal degradability of DM was calculated as A + [B(k_d_/(k_d_ + k_p_))], where k_d_ was the degradation rate and k_p_ was the fractional passage rate of the basal diet [20], as measured by the total ruminal evacuation technique. Bermudagrass hay data derived from the nonlinear model were analyzed with diet to consider a fixed effect, and period and cow were considered random effects using PROC GLIMMIX, as described previously. The LSMEANS option was used to generate individual treatment means. Significance was declared at *p* < 0.05, and tendencies were considered at 0.05 < *p* ≤ 0.10.

## 3. Results

### 3.1. Chemical Composition, Intake, and Digestibility

The chemical composition data are displayed in Table 1 and intake and digestibility data are displayed in Table 2. Hay DMI (kg/d and % BW) did not differ by diet (*p* > 0.14). As designed, supplement DMI (kg/d and % BW) was greater (*p* < 0.01) in medium-supplementation diets compared to low-supplementation diets, and all supplemented diets were greater (*p* < 0.01) than CON. Total DMI (kg/d) was greater (*p* ≤ 0.03) for MC and MH compared to LC and LH, which were not different from CON (*p* ≥ 0.10). Total DMI (% BW) was greater (*p* ≤ 0.05) from MH and MC compared to LH and CON, but LC was intermediate and not different (*p* ≥ 0.14) among supplemented diets. Total DMI (% BW) from all supplemented diets, except LH (*p* = 0.06), were greater (*p* ≤ 0.003) than CON. Total DM digestion % did not differ (*p* = 0.25) among diets. Digestible DMI (kg/d) from MH and MC was greater (*p* ≤ 0.04) than other diets; LC was greater (*p* = 0.01) than CON, but LH was intermediate and not different (*p* ≥ 0.08) from LC or CON. Digestible DMI (% BW) was greater (*p* < 0.01) in MH compared to LC, LH, and CON; MC was intermediate and not different (*p* ≥ 0.07) from MH or LC, and LH and CON were lesser (*p* ≤ 0.01) than all other diets, but not did not differ (*p* = 0.06) from each other. Dry-matter fill (kg), DM fill as a % of BW, passage rate (h^−1^), and retention time (h) did not differ among diets (*p* ≥ 0.31).

### 3.2. Fermentation

**Figure 1:**
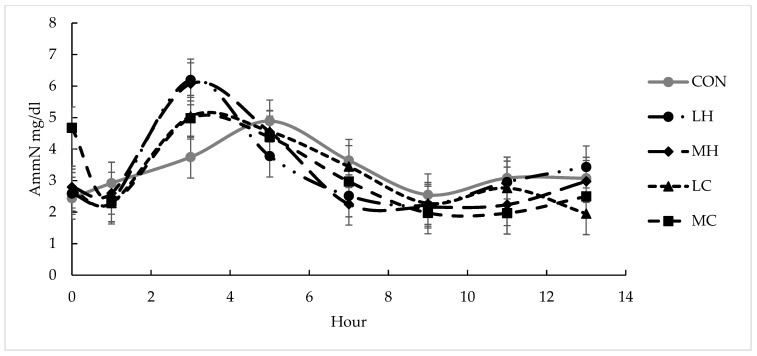
Ruminal ammonia-N concentrations (mg/dL) over time after supplement feeding from lactating beef cows offered bermudagrass hay supplemented with hominy feed or ground corn at two different rates of 0.25% of BW (DM basis) and 0.05% of BW (DM basis). CON = bermudagrass hay only (grey line with circle), LH = supplementation with hominy feed at 0.25% of BW (dotted and dashed line with circle), MH = supplementation with hominy feed at 0.50% of BW (dashed line with diamond), LC = supplementation with ground corn at 0.25% of BW (dashed line with triangle), MH = supplementation with hominy feed at 0.50% of BW (dashed line with square). There were significant effects for diet × time (*p* < 0.01) and time (*p* < 0.01). Error bars represented pooled standard error of the mean (SEM = 0.53).

**Figure 2:**
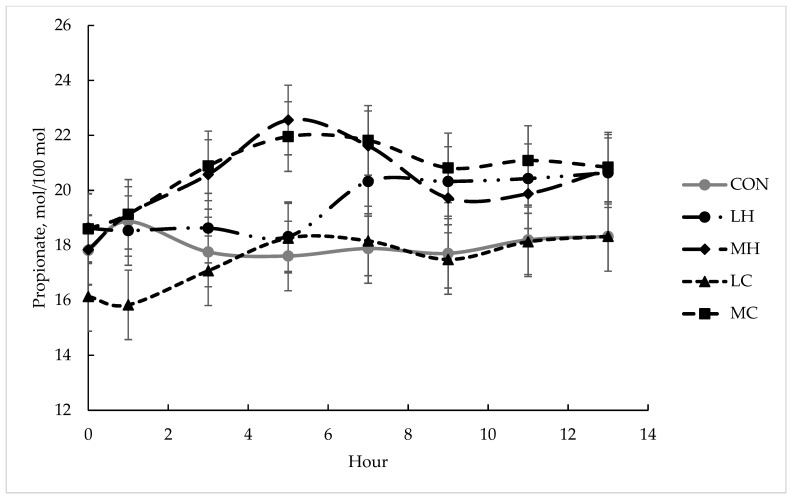
Propionate concentrations (mol/100 mol) over time after supplement feeding from lactating beef cows offered bermudagrass hay supplemented with hominy feed or ground corn at two different rates of 0.25% of BW (DM basis) and 0.05% of BW (DM basis). CON = bermudagrass hay only (grey line with circle), LH = supplementation with hominy feed at 0.25% of BW (dotted and dashed line with circle), MH = supplementation with hominy feed at 0.50% of BW (dashed line with diamond), LC = supplementation with ground corn at 0.25% of BW (dashed line with triangle), MH = supplementation with hominy feed at 0.50% of BW (dashed line with square). There were significant effects for diet × time (*p* = 0.02) and time (*p* < 0.01). Error bars represented pooled standard error of the mean (SEM = 1.11).

Fermentation data are presented in Table 3. A diet × time interaction was observed for ammonia-N (*p* < 0.01; Figure 1). The major difference in the trends observed was that ammonia-N concentrations peaked at 3 h post-feeding from cows offered the supplemented diets, but at 5 h post-feeding from cows that were offered CON. A trend for differences (*p* = 0.07) in pH for diets was observed and a time effect (*p* < 0.01) was observed. A time effect was detected for total VFA (*p* = 0.01), acetate (*p* = 0.05), butyrate (*p* < 0.01), and branched-chain fatty acids (*p* < 0.01), but diet did not impact these measurements (*p* ≥ 0.20). A diet × time interaction was detected for the propionate (*p* = 0.02; Figure 2). The general trend was for greater propionate concentrations from MH and MC than the other treatments between 3 and 7 h after feeding.

### 3.3. Hay In Situ

The hay in situ data are presented in Table 4. The water-soluble fraction (A), digestion lag time, and rate of digestion (k_d_) did not differ among diets (*p* > 0.13). The potentially degradable fraction (B) was greater (*p* ≤ 0.02) in LH compared to MH and CON, while LC and MC were intermediate and not different (*p* ≥ 0.06) from any other diets. The undegradable fraction (U) was greater (*p* < 0.002) in MH than LH and MC; CON and LC were greater (*p* ≤ 0.02) than LH, but not different (*p* ≥ 0.17) from MC or MH; and MC was not different (*p* ≥ 0.09) from CON, LC, or LH.

## 4. Discussion

### 4.1. Chemical Composition

The hominy feed used in this study was lower in starch and greater in NDF, compared to descriptions provided by NASEM [3], in which, NASEM lists hominy feed as 167.9 g/kg NDF, 567.7 g/kg starch, 71.5 g/kg fat, and 102.7 g/kg CP. As a co-product, nutritional characteristics for HF can be variable [21]. Hominy most notably differed from GC by having lower starch, and greater NDF and fat content. Similar trends were noted by Cooke et al. [8]; starch levels for GC and HF (641 and 472 g/kg starch, respectively), were greater, but differences between the feeds was similar to the current study. Boyd et al. [7] observed few differences between GC and HF, including starch values of 611 in GC and 585 g/kg in HF. However, GC used in the current study had greater NDF and CP compared to other studies [7,8]. Cooke et al. [8] noted CP levels of 101 g/kg and NDF of 149 g/kg, while Boyd et al. [7] noted CP of 91 g/kg and NDF of 128 g/kg. Bermudagrass hay had 707 g/kg NDF and the CP level of 103 g/kg and met CP requirements for lactating beef cows [3].

### 4.2. Intake and Digestibility

Hay intake, either as kg DM or % BW, was not reduced with supplementation. Concentrate supplementation on high-forage diets may result in the substitution of hay intake for supplement intake. Loy et al. [22] observed a reduced forage intake by heifers offered a grass hay-based diet (82 g/kg CP) supplemented with DDGS and dry-rolled corn at 0.40% BW. Sanson et al. [23] observed reduced native grass hay (95 g/kg CP) intake by steers with both barley and dry-rolled corn with increasing supplementation rates from 0, 0.25, to 0.50% of BW. Intake reductions were also observed when DDGS at 0.50% was supplemented to lactating beef cows consuming bermudagrass (170 g/kg CP) [10].

Total DMI (kg/d) in the present study was either unchanged at the 0.25% supplementation level or increased at the 0.50% supplementation level compared with CON. Total DMI (% BW) was greater than CON from all supplementation diets except LH, which was not different from LC or CON. This scenario is the ideal situation where feeding supplements add to the total DMI, rather than substituting supplement for hay. Moore et al. [24] reviewed the effects of supplementation on voluntary forage intake and determined that forage organic matter (OM) intake was reduced with supplementation when the forage total digestible nutrients (TDN):CP ratio was below 7 (adequate protein), but that forage OM intake increased with supplementation when the forage TDN:CP ratio was above 7 (protein deficit). Furthermore, source of supplementation had little impact on the relationship between OM intake and the TDN:CP ratio. With the CP concentration in the hay in the present study (103 g/kg DM), the TDN:CP ratio would be well below 7, which would lead to expectations of a reduction in forage OM intake.

Dry matter digestibility was 53% for CON, combined with the CP levels 103 g/kg, which indicates that the bermudagrass was of medium quality according to Leng [25]. Galloway et al. [26] observed digestible OM levels for un-supplemented bermudagrass of 52.7%, while Nieman et al. [10] observed digestibility levels for bermudagrass of 63.7%. No differences in DM digestibility were observed among treatments, however. Similarly, Cooke et al. [8] did not observe differences in DM or OM digestibility for dairy cows supplemented with GC or HF. Though NDF digestibility was not measured in the current study, Larson et al. [4] observed greater NDF digestibility in high-concentrate, beef-finishing diets with HF supplementation, compared to GC, which was attributed to the greater digestible NDF in HF and lesser starch reducing the associative effects. Contrary to Larsen et al. [4], Cooke et al. [8] observed increased NDF digestibility with GC supplementation, compared to HF supplementation on dairy diets. Digestible DMI (kg/d) was similar within the supplementation level (0.25 or 0.5% BW) between GC and HF in the present study. Digestible DMI (% BW) was greater from LC, MC, and MH, compared with CON, and LH was the same as CON. The higher than usual NDF and CP concentrations in GC that were also similar to levels in HF, likely contributed to a few differences in DM digestibility resulting from the lack of associative effects.

No differences were observed in DM fill, passage rate, or retention time. Greater values for passage rates (ranging from 4.18–4.80% h^−1^) for bermudagrass-based diets un-supplemented or supplemented with GC or soy hulls, at levels ranging from 0.25% to 0.7%, were observed by Galloway [26]. Retention times did not differ among treatments, but values were greater than those of stocker calves grazing on bermudagrass (20.5, h, [27]). A lack of response in rumen-retention time is not unexpected, as rumen-retention time is influenced by intake with a linear relationship between increasing intake and decreasing retention time [28], and intake differences were not greatly different in the current study.

### 4.3. Fermentation

Ammonia-N was affected by a diet × time interaction in this study. Supplement types were closely paired and followed similar patterns over the 13 h sampling period, with the greatest peak at 3 h, though the peak was lower for GC than HF diets. The numerically lower peak for GC diets was likely due to the greater amount of starch offered by GC compared to HF. The rapid digestion of starch provided increased energy availability for the increased utilization of ammonia-N and microbial–protein synthesis in the rumen [29], thus reducing ammonia-N concentrations, noticeably around 3 h, compared to HF. Greater soluble protein in HF may have also contributed to greater ammonia-N in HF diets, as HF contains more soluble protein than GC [30]. Ammonia-N levels for the un-supplemented diets did not peak until 5 h, and the peak for CON averaged 4.89 mg/dL; similar to 4.71 mg/dL for GC at 3 h, while HF averaged 6.13 mg/dL at 3 h. The later peak in ammonia-N in CON was likely related to the lag in digestion that occurred on a forage only diet, while both HF and GC were more rapidly degraded. Sanson et al. [23] observed a quadratic relationship for ammonia-N with supplementation rates of 0, 0.25% BW, and 0.50% BW in steers consuming native grass hay (95 g/kg CP), indicating lower ammonia-N at higher supplementation rates for GC. Ammonia-N concentrations were lower in supplemented diets compared to the control, and more so with the greater supplementation rate (0.50% BW). Values for ammonia-N were similar to those reported by Sanson et al. [23], in which, the un-supplemented treatment averaged 3.62 mg/dL; GC at 0.25% BW averaged 4.65 mg/dL; and GC at 0.50% BW averaged 2.86 mg/dL, whereas, barley at 0.25% BW averaged 3.59 mg/dL and 0.50% BW averaged 3.46 mg/dL.

Trends were observed for pH, both for the interaction of diet × time and for diet. In the present study, the highest pH values were from CON and LC and lowest from LH, MC, and MH. Lower ruminal pH was expected in GC diets, as reduced pH has been observed in forage-based diets with 0.25 and 0.50 % of BW supplementation with GC due to the rapidly degrading starch [23]. Loy et al. [22] also observed reductions in rumen pH compared to the control in heifers consuming dry-rolled corn, which was fed daily, dried-rolled corn fed every other day, DDGS fed daily, and DDGS fed every other day; although, they did not observe differences between DDGS and dried-rolled corn. Potentially, lower starch and greater NDF in the corn utilized in this study prevented significant reductions in pH. Time effects were observed for total VFA concentration and concentrations of acetate, butyrate, and branched-chain fatty acids, but no diet effects were detected. Sanson et al. [23] also did not detect differences in acetate for steers fed medium-quality hay and different levels of GC and barley. Volatile fatty-acid production did not differ between HF and GC in an in vitro study, indicating the similarity of the supplements [31]. A diet × time interaction was observed for propionate. Propionate was greater for both MH and MC at hour 5, compared to LH and LC and CON. Greater amounts of fermentable components of both GC and HF at the higher supplementation rate provided more substrate for propionate-producing bacteria. Loy et al. [22] also observed an increase in propionate for supplemented treatments (dry-rolled corn and DDGS fed at 0.40% BW daily or 0.80% BW every other day), compared to the control. Sanson et al. [23] did not observe differences in propionate for diets at different rates (0.25% and 0.50% of BW) or supplements (GC and barley).

### 4.4. Hay In Situ

Only the B fraction and U fraction differed among diets. Differences for the B or U fraction do not appear to be based on supplement type or rate. The B fraction was only greater from LH when compared with CON, and was not different among the other diets, and the U fraction was greater from MH than MC and LH. We would expect supplementation practices, that impact the rumen negatively, would reduce the B fraction, but this did not appear to be the case in the present study. Therefore, the modest changes in ruminal pH did not impact the degradable fraction or the degradation rate of the bermudagrass hay. In cattle-finishing diets, Larson et al. [4] concluded that that the greater fiber digestibility of HF, reduced the negative, associate effects of starch digestion on fiber digestion in the rumen, compared to GC. Cooke et al. [8] also observed HF appeared to support greater digestibility of DM compared with GC as the proportion of ryegrass (as opposed to corn silage) increased in the diet. In the current study, there does not appear to be consistent advantages to bermudagrass disappearance with HF or GC supplementation, but digestibility was not reduced by supplementation, and therefore, did not result in negative, associative effects.

## 5. Conclusions

Hominy feed or ground corn can be fed at levels up to 0.50% BW as a supplement for lactating beef cows consuming medium-quality bermudagrass hay without reducing intake or digestibility. Digestible dry-matter intake was increased with both ground corn and hominy feed when fed at levels up to 0.50% BW, thereby potentially increasing the energy status of those cows. Bermudagrass degradability was not reduced with supplementation of either supplement type or feeding level and ruminal measurements were similar for both levels of hominy feed and ground corn supplementation. Neutral detergent fiber and crude-protein levels were unusually high in the ground corn used in the present study, which may have contributed to the lack of associate effects detected at 0.50% BW that have been noted in other studies. Hominy feed can be used as an alternative feed to ground corn as an energy supplement without creating negative effects on measurements that are potential indicators of animal performance.

## Figures and Tables

**Table 1:** Chemical composition of bermudagrass hay and supplements offered to lactating, ruminally canulated cows (dry-matter basis).

Item ^1^	Bermudagrass Hay	Corn	Hominy
	g/kg DM		
CP	103	123	113
Starch	34	574	417
NDF	707	160	275
ADF	324	43	70
Ash	80	25	30
Ether extract	10	40	70

^1^ DM = dry matter; CP = crude protein; NDF = neutral-detergent fiber; ADF = acid-detergent fiber.

**Table 2:** Intake, digestibility, and ruminal DM fill by lactating, ruminally cannulated beef cows offered medium quality bermudagrass hay and supplemented with either corn or hominy.

Item ^1^	CON ^2^	LC	MC	LH	MH	SEM	*p*-Value
Hay DMI, kg/d	14.3	13.9	13.6	13.5	13.5	0.75	0.35
Hay DMI, % BW	2.38	2.41	2.27	2.30	2.27	0.152	0.14
Supplement DMI, kg/d	0.00 c	1.29 b	2.66 a	1.38 b	2.73 a	0.119	<0.01
Supplement DMI, % BW	0.00 c	0.22 b	0.44 a	0.23 b	0.45 a	0.005	<0.01
Total DMI, kg/d	14.3 b	15.2 b	16.3 a	14.9 b	16.3 a	0.78	<0.01
Total DMI, % BW	2.38 c	2.63 ab	2.71 a	2.53 bc	2.72 a	0.152	<0.01
DM digestion, %	53.0	54.7	54.4	53.0	56.3	2.10	0.25
Digestible DMI, kg/d	7.6 c	8.3 b	8.9 a	7.9 bc	9.2 a	0.52	<0.01
Digestible DMI, % BW	1.27 c	1.44 b	1.47 ab	1.34 c	1.54 a	0.107	<0.01
DM fill, kg	13.7	13.2	14.5	12.9	14.2	0.65	0.31
DM fill, % BW	2.30	2.30	2.43	2.15	2.39	0.154	0.47
Passage rate (k_p_), h^−1^	0.037	0.039	0.035	0.040	0.036	0.0034	0.50
Retention time, h	27.7	26.2	28.8	25.3	29.6	2.62	0.41

^1^ MH = hominy feed offered at 0.5% of BW; DMI = dry-matter intake; BW = body weight. ^2^ CON = control, no supplement; LC = corn offered at 0.25% of cow BW; MC = corn offered at 0.5% of cow BW; LH = hominy feed offered at 0.25% of cow BW; MH = hominy feed offered at 0.5% of BW. a, b, c Means within a row without a common letter designation differ (*p* < 0.05).

**Table 3:** Ruminal fluid characteristics ^1^ of lactating, ruminally cannulated beef cows offered medium quality bermudagrass hay supplemented with either corn or hominy.

Item	CON ^2^	LC	MC	LH	MH	SEM	Diet	Time	Diet × Time
Ammonia-N, mg/dL	3.3	3.2	3.2	3.5	3.2	0.53	0.99	<0.01	<0.01
pH	6.4	6.4	6.2	6.1	6.2	0.15	0.07	<0.01	0.08
Total VFA ^3^, mM	106.3	99.5	108.2	107.2	105.7	5.21	0.77	<0.01	0.40
Acetate, mol/100 mol	75.00	69.1	72.9	73.1	71.6	3.5	0.78	0.05	0.54
Propionate, mol/100 mol	18.0	17.4	20.7	19.5	20.3	1.11	0.20	<0.01	0.02
Butyrate, mol/100 mol	10.8	10.6	11.9	12.1	11.4	0.73	0.47	<0.01	0.16
Branched-chain VFA ^4^, mol/100 mol	2.5	2.3	2.7	2.5	2.5	0.16	0.58	<0.01	0.11

^1^ Rumen-fluid samples were collected immediately prior to feeding and 1, 3, 5, 7, 9, 11 and 13 h after feeding. Means in this table represent the average values across all sampling times. ^2^ CON = control, no supplement; LC = corn offered at 0.25% of cow BW; MC = corn offered at 0.5% of cow BW; LH = hominy feed offered at 0.25% of cow BW; MH = hominy feed offered at 0.5% of BW. ^3^ VFA = volatile fatty acids. ^4^ Branched-chain, volatile fatty acids (VFA) represents the total of concentrations of isobutyrate, valerate, and isovalerate.

**Table 4:** In situ DM disappearance in lactating, ruminally cannulated beef cows offered medium quality bermudagrass hay and supplemented with different levels of corn or hominy.

In Situ Parameter	CON ^1^	LC	MC	LH	MH	SEM	*p*-Value
Water-soluble fraction (A), %	21.9	21.7	21.8	22.0	22.1	0.92	0.79
Digestion lag time (lag), h	1.06	0.76	2.00	2.44	2.29	0.722	0.13
Potentially degradable fraction (B), %	47.9 b	48.6 ab	49.2 ab	50.5 a	47.1 b	0.96	0.04
Rate of digestion (k_d_), h^−1^	0.03	0.03	0.03	0.03	0.03	0.003	0.24
Undegradable fraction (U), %	30.2 ab	29.7 ab	29.0 bc	27.5 c	30.8 a	0.94	0.01
Effective degradability ^2^, %	44.7	43.6	44.2	42.5	45.1	1.56	0.39

^1^ CON = control, no supplement; LC = corn offered at 0.25% of cow BW; MC = corn offered at 0.5% of cow BW; LH = hominy feed offered at 0.25% of cow BW; MH = hominy feed offered at 0.5% of BW. ^2^ Effective ruminal degradability of DM was determined as A + [B(k_d_/(k_d_ + k_p_))], where k_p_ is the fractional-passage rate of the basal diet. a, b, c Means within a row without a common letter designation differ (*p* < 0.05).

## Data Availability

Data will be made available in the USDA National Agricultural Library upon publication.
